# Risk Factors for Overweight and Obesity among Thai Adults: Results of the National Thai Food Consumption Survey

**DOI:** 10.3390/nu20100060

**Published:** 2010-01-21

**Authors:** Nattinee Jitnarin, Vongsvat Kosulwat, Nipa Rojroongwasinkul, Atitada Boonpraderm, Christopher K. Haddock, Walker S.C. Poston

**Affiliations:** 1 Institute for Biobehavioral Health Research, NDRI-Mid America, 1920 W. 143^rd^ Street, Suite 120, Leawood, Kansas 66224 USA; Email: haddock@ndri.org (C.K.H.); poston@ndri.org (W.S.C.P); 2 Mead Johnson Nutrition (Thailand) Ltd., 388 Exchange tower 14^th^ Fl., Sukhumvit Road, Klongtoey, Bangkok, 10110 Thailand; Email: vongsvat.kosulwat@mjn.com; 3 Institute of Nutrition, Mahidol University, Phutthamonthon 4 Rd., Salaya, Phutthamoonthon, Nakorn Pathom, 73170 Thailand; Email: nunrr@mahidol.ac.th (N.R.); nunrr@mahidol.ac.th (A.B.)

**Keywords:** overweight/obesity, SES, smoking, dietary intake, Thailand

## Abstract

We evaluated the associations between overweight and obesity and socio-economic status (SES), behavioral factors, and dietary intake in Thai adults. A nationally representative sample of 6,445 Thais adults (18-70 years) was surveyed during 2004-2005. Information including demographics, SES characteristics, dietary intake, and anthropometrics were obtained. Overall, 35.0% of men, and 44.9% of women were overweight or obese (BMI ≥ 23 kg/m^2^) using the Asian cut-points. Regression models demonstrated that age was positively associated with being overweight in both genders. In gender-stratified analyses, male respondents who were older, lived in urban areas, had higher annual household income, and did not smoke were more likely to be classified as overweight and obese. Women who were older, had higher education, were not in a marriage-like relationship and were in semi-professional occupation were at greater risk for being overweight and obese. High carbohydrate and protein intake were found to be positively associated with BMI whereas the frequent use of dairy foods was found to be negatively associated with BMI among men. The present study found that SES factors are associated with being classified as overweight and obese in Thai adults, but associations were different between genders. Health promotion strategies regarding obesity and its related co-morbidity are necessary.

## 1. Introduction

Overweight and obesity have been considered a serious health problem worldwide since 1997 [1]. Both developed and developing countries are experiencing increasing rates of overweight and obesity. The new WHO report indicated that 1.6 billion adults were overweight and more than 400 million adults were obese, and at least 20 million children under 5 years were overweight [2]. Similar trends showing increasing overweight and obesity prevalence also can be seen in Thailand. Data from the Thailand National Health Examination Survey (2003-2004) revealed a significant increase in the prevalence of overweight and obesity, from 25% in 1991 to 48% in 2004 in a sample of Thai adults aged 35-59 years [3]. 

There have been a number of studies examining the risk factors contributing to the prevalence of overweight and obesity. Numerous studies conducted in developed countries have found an association between socio-economic status (SES) and overweight and obesity, with lower SES individuals experiencing greater risk for overweight and obesity than those in higher SES [4,5,6,7]. However, few nationally representative studies in developing countries, particularly Thailand, have examined what factors increase risk for overweight and obesity and, in particular, whether SES is an independent risk factor [8,9].

Thailand, in particular, has experienced significant economic and health transitions for more than two decades. Its structure has gradually changed from a traditional agricultural setting to an industrialized structure and from a primarily rural population to an urbanized community. Consequently, Thai lifestyles, such as diet and activities, also have changed. The dietary intake pattern changed from traditional high-carbohydrate diets, which rely heavily on rice and vegetables, to diets high in fat and sugar. In addition, the pattern of food expenditure changed from purchasing fresh foods for home preparation to purchasing ready-to-eat highly processed foods [10]. During the same period, occupational and commuting physical activity have progressively declined because of an increase in urbanization, industrialization, and automation, resulting in increased time spent in sedentary activities [11,12]. 

Therefore, better understanding the relationship between SES pattern, behavioral factors, dietary intake and overweight and obesity prevalence in the Thai population is considered necessary. The aim of this study was to address and examine the relationship of socioeconomic status, behavioral factor, dietary pattern on overweight and obesity prevalence in a nationally representative sample of Thai adults. The specific interest was in determining which indicator most strongly influenced overweight and obesity in this population. 

## 2. Results and Discussion

### 2.1. Sample Size and Characteristics

Sample means and proportions of the characteristics of a representative sample of Thai adults were calculated separately for men and women (see Table 1). The sample was comprised of 50.8% (n = 3275) females and 49.2% (n = 3170) males aged 18 to 70 years old. Of the total sample, 35.0% of males and 44.9% of females were overweight (BMI ≥ 23.0 kg/m^2^). The mean BMI was significantly higher among women (23.1 ± 4.5 kg/m^2^) than men (22.1 ± 3.4 kg/m^2^), *p* < 0.001. In addition, men tended to engage in more smoking and drinking behaviors than observed in women (*p* < 0.001 for both behaviors). When considering dietary intake, men significantly consumed more total energy and macronutrients, more servings of rice and meat, but fewer servings of dairy products than women did. 

**Table 1:** Characteristics of a representative sample of Thai adults by gender^a^.

Variables	Men (n = 3,170)	Women (n = 3,275)	p- value
Age (years)	40.7 ± 17.2	40.8 ± 16.6	0.885
BMI (kg/m^2^)	22.1 ± 3.4	23.1 ± 4.5	<0.001
Overweight, BMI ≥ 23.0 kg/m^2^	35.0 (33.3, 36.7)	44.9 (43.2, 46.6)	<0.001
Education Levels			<0.001
Basic	53.2 (51.5, 55.0)	59.3 (57.6, 61.0)	
Secondary	39.0 (37.3, 40.7)	32.1 (30.5, 33.7)	
High	7.8 (6.8, 8.7)	8.6 (7.6, 9.6)	
Places of Residence			0.921
Rural	43.8 (42.1, 45.6)	43.9 (42.2, 45.6)	
Urban	56.2 (54.5, 57.9)	56.1 (54.4, 57.8)	
Annual Household income ($)	3332.4 ± 3134.0	3212.3 ± 3130.2	0.130
Currently Smoking (%)	43.0 (41.3, 44.7)	3.8 (3.1, 4.4)	<0.001
Any Alcohol Consumption (%)	11.5 (10.4, 12.6)	1.0 (0.7, 1.4)	<0.001
Dietary Daily Intake			
Total Energy (kcal)	1597.4 ± 636.0	1320.0 ± 556.6	<0.001
Carbohydrate (g)	241.2 ± 110.7	199.1 ± 93.6	<0.001
Protein (g)	60.97 ± 28.5	51.4 ± 26.4	<0.001
Fat (g)	40.4 ± 27.1	35.1 ± 24.4	<0.001
Food Groups (serving sizes)			
Rice and Starchy Foods	9.5 ± 5.7	8.0 ± 4.7	<0.001
Vegetables	6.2 ± 5.2	6.1 ± 5.0	0.549
Fruits	5.0 ± 4.8	5.0 ± 4.3	0.512
Dairy	0.3 ± 0.5	0.4 ± 0.7	<0.001
Meat	13.2 ± 11.6	11.4 ± 9.7	<0.001

^a^Values are means ± SD or proportions (95% confidence interval), as appropriate for the variables. *P-values* for the difference in variables based on chi-square test or an independent sample t-test as appropriate.

### 2.2. Characteristics of Participants Stratified by Gender and BMI Status

Table 2 presents the characteristics of the study participants by gender and by BMI status. In both genders, those who were overweight were approximately eight years older than those who were not (*p* < 0.001). Although there were differences in overweight and obesity prevalence based on place of residence, only men showed a significant difference. In both genders, the percentage of overweight was higher in urban areas than those in rural areas (*i.e.*, 62.0% and 56.0% for men and women, respectively). There also was a statistically significant association between BMI status and socioeconomic variables in both genders (see Table 2). 

**Table 2:** Characteristics of a representative sample of Thai adults by gender and BMI status.

Variables	Men (n = 3,170)		Women (n = 3,275)	
	BMI < 23.0 (n = 2,056)	BMI ≥ 23.0 (n = 1,117)	BMI < 23.0 (n = 1,794)	BMI ≥ 23.0 (n = 1,463)
Age (years)	38.8 ± 17.5	44.2 ± 15.8***	37.0 ± 17.0	45.4 ± 14.9***
Places of Residence	χ^2^ = 22.9***		χ^2^ = 0.002	
Rural	964 (46.9)	421 (38.0)	791 (44.1)	644 (44.0)
Urban	1092 (53.1)	686 (62.0)	1003 (55.9)	819 (56.0)
Education Levels	χ^2^ = 9.8**		χ^2^ = 0.02***	
Basic	1048 (51.2)	630 (57.0)	849 (47.5)	1076 (73.6)
Secondary	832 (40.6)	399 (36.0)	731 (40.9)	313 (21.4)
High	168 (8.2)	77 (7.0)	208 (11.6)	73 (5.0)
Employment Status	χ^2^ = 3.1		χ^2^ = 5.1	
Employed	1435 (82.2)	875 (82.9)	996 (67.0)	885 (63.0)
Retired	99 (5.7)	71 (6.7)	77 (5.2)	85 (6.1)
Unemployed	212 (12.1)	109 (10.3)	413 (27.8)	434 (30.9)
Annual Household income ($)	3095.1 ± 2924.2	3775.0 ± 3451.8***	3145.3 ± 3032.1	3299.4 ± 3255.9
% Tobacco Smoking	928 (45.1)	431 (38.9)***	69 (3.8)	53 (3.6)
Dietary Daily Intake				
Total Energy (kcal)	1570.4 ± 619.4	1644.0 ± 660.8***	1314.8 ± 558.2	1326.3 ± 555.8
Carbohydrate (g)	237.8 ± 107.6	247.1 ± 115.4*	196.9 ± 92.6	201.9 ± 94.9
Protein (g)	59.6 ± 27.5	63.5 ± 30.2***	51.1 ± 25.9	51.6 ± 26.9
Fat (g)	39.5 ± 26.5	41.9 ± 28.3*	35.5 ± 24.2	34.5 ± 24.8
Food Groups (serving sizes)				
Rice and Starchy Foods	9.5 ± 5.7	9.7 ± 5.6	7.8 ± 4.6	8.3 ± 4.8**
Vegetables	6.1 ± 5.1	6.3 ± 5.3	5.8 ± 4.6	6.5 ± 5.3***
Fruits	5.0 ± 4.9	4.8 ± 4.5	5.2 ± 4.5	4.9 ± 4.2
Dairy	0.3 ± 0.6	0.2 ± 0.4***	0.5 ± 0.7	0.4 ± 0.6***
Meat	13.2 ± 11.5	13.2 ± 11.9	11.5 ± 10.0	11.3 ± 9.3

*** *p* < 0.001; ** *p* < 0.01; * *p* < 0.05

Among men, there were significant differences based on BMI status on education levels (χ^2^(2) = 9.8, *p* < 0.01) and annual household income (*F*(3066) = 36.0, *p* < 0.001). However, a chi-square test revealed that only education was associated with BMI status for women (χ^2^(2) = 0.002, *p* < 0.01). Smoking was more common in men who were not overweight (*p* < 0.001). For dietary intake, only men showed statistically significant differences, indicating that overweight men consumed more total energy, carbohydrate, protein, and fat than those who were not overweight. When considering eating patterns among men, total grams of food consumed were not different between overweight and non-overweight groups. Among women, consumption of rice and starchy foods (*p* < 0.01), and vegetables (*p* < 0.001) were significantly higher in overweight participants than their non-overweight counterpart. In both genders, lower dairy product consumption was observed among overweight participants (*p* < 0.001). 

### 2.3. Logistic Regression Models on the Likelihood of Being Overweight

Table 3 and Table 4 show the multinomial regression results with odd ratios and their 95% confidence intervals for males and females, respectively. Separate models were used for men and women, and potential confounders such as age and marital status were controlled. Results from the initial Univariate analysis showed that men and women had different patterns of odd ratios for SES variables and dietary consumptions. Multivariate logistic regression analyses revealed similar patterns of risk for overweight and obesity for males and females based on age. Risk for overweight and obesity was greater for men and women aged older than 25 years. Interestingly, participants aged 46-55 years had the highest risk of being overweight and obese, compared to those aged under 25 years (OR=2.6; 95% CI=1.8-3.7 for men; OR=4.8; 95% CI =3.0-7.6 for women). Our findings also indicated that male respondents with higher income or those who had annual household income more than USD $3875.03 were 1.8 times (95% CI: 1.3-2.4) at risk of being overweight and obese than those who in the lowest quartile. 

**Table 3:** Logistic Regression Models on the Likelihood of Being Overweight (Odd Ratios (OR) and their 95% Confidence Intervals (CI) in Men.

Characteristics	Men (n = 3,170)	
	OR	95% CI
Age		
18-25	1.0	
26-35	1.8	1.3, 2.5***
36-45	2.3	1.6, 3.2***
46-55	2.6	1.8, 3.7***
56-65	2.4	1.6, 3.5***
66+	1.9	1.2, 2.9**
Place of Residents		
Rural	1.0	
Urban	1.3	1.1, 1.6**
Occupational Status		
Manual	1.0	
Routine Non-manual	1.6	1.3, 2.0***
Semi-professional	0.9	0.5, 1.6
Managers & Professionals	1.2	0.7, 1.9
Annual Household Income		
Quartile 1	1.0	
Quartile 2	1.4	1.1, 1.7*
Quartile 3	1.8	1.3, 2.4***
Tobacco Smoking		
No	1.0	
Yes	0.7	0.6, 0.8***
Carbohydrate Intake		
Less than 300 g (100%)	1.0	
300-450 g (150%)	1.5	1.1, 1.9**
450-600 g (200%)	0.9	0.6, 1.4
More than 600 g (> 200%)	1.6	0.8, 3.2
Protein Intake		
Less than 50 g (100%)	1.0	
50-75 g (150%)	1.1	0.9, 1.6
75-100 g (200%)	1.4	1.0, 2.1*
More than 100 g (> 200%)	1.6	1.1, 2.7*
Dairy Consumption		
1-2 portions (as recommendation)	1.0	
3-5 portions (twice of the recommendation)	0.6	0.4, 1.0*
More than 5 portions (more than twice)	0.7	0.3, 1.8

*** *p* < 0.001; ** *p* < 0.01; * *p* < 0.05

When considering other SES status and lifestyle factors, place of residence, occupational level and smoking status significantly predicted overweight and/or obesity in male participants (Table 3). Men who lived in urban areas were 1.3 times (95% CI = 1.1-1.6) and those who worked non-routine manual jobs were 1.6 times (95% CI = 1.3-2.0) more likely to be overweight and/or obese, but smokers were at significantly lower risk of being overweight and/or obese versus healthy weight compared to non- or ex-smokers (OR = 0.7; 95% = CI: 0.6-0.8). For females, education level, marital status, and occupation were the stronger predictors of overweight and obesity (see Table 4). Risk for overweight or obesity was greatest for females who were not married (OR = 1.6; 95% CI = 1.2-2.1), and in semi-professional occupations (OR = 3.3; 95% CI = 1.0-11.4), but was lowest among those who had higher education (OR = 0.5; 95% CI = 0.3-0.9).

Macronutrient intake was significantly associated with higher risk of overweight and obesity only in male participants, with those who consumed 300-450 g per day of carbohydrate (150% of Thai recommendation) having 1.5 times (95% CI = 1.1-1.9) the risk of obesity as those who consumed less than 300 g (as the recommendation). In addition, participants who reported consuming more than 75g per day of protein (more than 200% of Thai recommendation) experienced a 40-60% (95% CI = 1.0-2.1, 1.1-2.7) higher risk of being overweight compared to those who follow the Thai daily recommendation (Table 3). Moreover, among those who had dairy 3-5 portions per day (2 times of the recommendation) had lower risk of being overweight or obese (OR = 0.6; 95% CI = 0.4-1.0).

**Table 4:** Logistic Regression Models on the Likelihood of Being Overweight (Odd Ratios (OR) and their 95% Confidence Intervals (CI) in Women.

Characteristics	Women (n = 3,275)	
	OR	95% CI
Age		
18-25	1.0	
26-35	1.8	1.2, 2.7**
36-45	2.5	1.6, 3.9***
46-55	4.8	3.0, 7.6***
56-65	2.9	1.8, 4.8***
66+	2.1	1.2, 3.8**
Education Levels		
Basic	1.0	
Secondary	0.6	0.5, 0.9**
High	0.5	0.3, 0.9*
Marital Status		
Married	1.0	
Others	1.6	1.2, 2.1***
Occupational Status		
Manual	1.0	
Routine Non-manual	1.2	1.0, 1.5
Semi-professional	3.3	1.0, 11.4*
Managers & Professionals	0.5	0.2, 1.1

*** *p* < 0.001; ** *p* < 0.01; * *p* < 0.05

### 2.4. Discussion

The present data from the TFCS showed a significant association between a number of risk factors and overweight and obesity in Thai samples. Separated by genders, male respondents who were older, lived in urban areas, had higher annual household income, and who were a non- or former smoker were identified to be at increased risk for overweight and obesity. In addition, female participants who were older, had higher education, were not in a marriage-like relationship, and were in semi-professional occupation were at greater risk for being overweight and obesity. 

#### 2.4.1. Demographic Factors

Men and women who were older were more likely to be overweight and obese than were those with younger age. This observation is in line with results of other studies showing similar associations between age and overweight and obesity [13]. The present finding also showed that participants aged between 46-55 years old had the highest risk of being overweight and/or obese, which might be due to the weight gain from life transitions during that time such as retirement [14,15] or menopause [16,17,18]. Marital status appears to be a strong predictor only in female participants. Women who were not in a marriage-like relationship or living with a partner were at increased risk of being overweight and obese. The findings support some studies [13,19] that demonstrated that marriage was associated with weight loss and low BMI, but were contradicted by several studies, which found an association between obesity and being and getting married [20,21,22]. For this sub-sample, their partners might provide support in terms of healthy lifestyles, *i.e.*, eating healthy food and/or being involved in physical activity. In addition, non-married individuals are more independent than married couples, which could have been more associated with an unhealthy lifestyle [23]. Unfortunately, we did not have in-depth information regarding how partners’ influence on participant health behaviors. More research is necessary to determine the relationship between married individuals and health behaviors. 

Place of residence was a significant predictor of overweight and obesity in males but not females. Urban male residents have a greater risk for overweight and obesity than those who lived in rural area. This is discordant with past studies, which found an association between being overweight and/or obese with living in rural areas [24,25]. However, this finding was similar to other studies using Thai samples indicating that residing in urban areas was associated with higher prevalence of overweight and obesity [8,9]. Several explanations have been examined for the association between residential area and overweight/obesity prevalence. The effect of urbanization might be one explanation for overweight and obesity prevalence inequalities [12,26]. People in urban areas experience more sedentary lifestyle, less physical activity, and changes in dietary pattern than those in rural areas, which might account for the obesity trend in urban residents. 

#### 2.4.2. SES Factors

In this present study, SES was measured via occupational status, annual household income, and education. The pattern of association between occupational status and overweight or obesity was observed in both genders, although it was a nonlinear relationship. Those with lower job status had lower risk of overweight and obesity. These findings were discordant with previous studies indicating that a low occupational level is related to high overweight and obesity prevalence due to high work stress, low job control, and less leisure-time and physical activity [27,28,29]. However, in Thailand, low status jobs, such as farming or construction, are more physically demanding and involve heavy manual labor, which could decrease risk for overweight and obesity, while high-status jobs are associated with more sedentary behaviors, which could be a possible explanation for the association between occupational level and overweight in these sub-samples. Therefore, further research focusing on occupational activities, such as sitting time, leisure-time activity, and physical activity is needed. 

Our study also found associations between SES indicators and obesity. Overweight and obesity in men was positively associated with annual household income, while female overweight and obesity was negatively associated with level of education. This finding is supported by the other studies [4,30,31,32]. In addition, Sobal and Sunkard [4], and Popkin *et al.* [33] suggested that in developing countries, income strongly influences risk of obesity whereas education might be a protective factor against obesity. It also is noteworthy that there are gender differences in the relation between SES and being overweight and/or obese in this sample. Differences in body shape perceptions and attitudes between men and women could provide alternative explanations [34,35]. Furthermore, overweight and obesity stigma from public and social pressure on weight status might play an important role in differences between women and men, which tend to result in women having greater dietary restriction [36,37]. Although, the current study did not demonstrate a strong relationship between SES and overweight and obesity, levels of education and household income are evidently considered as powerful predictors for overweight and obesity in this sample. 

#### 2.4.3. Behavioral Factors

The association between behavioral factors and risk of overweight and obesity was observed only in male participants. Smoking was found to be a strong predictor for lower body weights among men. Male smokers were less likely to be overweight or obese compared to males who did not smoke. The data on smoking and body weight support the results of other studies indicating that smoking is consistently associated with lower body mass [38,39,40]. However, smoking should not be used as an alternative approach for weight management because of its substantial negative health consequences. Among female participants, smoking was not associated with weight status. However, the small sample size of female smokers (*i.e.*, 122 smokers among 3,275 women) limited statistical power and ability to detect differences in the various study outcomes among women. In addition, smoking could be underreported by the Thai women in our sample. Unlike Western countries, female smoking is not well accepted in Asian societies and it is not culturally appropriate for women to admit to smoking, mainly because of a socio-cultural belief and social norms [41,42]. 

#### 2.4.4. Dietary Intake

The relationship between dietary intake and obesity was considered in the current study. Although dietary factors were weakly associated with risk of being overweight and obese, carbohydrate and protein intake was found to be positively associated with overweight and obesity, and the frequent consumption of dairy products was found to be negatively associated with greater BMI among men. However, this finding is inconsistent with previous studies supporting the negative association between carbohydrate and protein intake and BMI [43,44], and positive associations between dairy foods and BMI [45]. The differences between other studies and the present study may result in part from cultural, environmental and behavioral differences such as specific food choices, food norms, food availability, and food diversity. Therefore, further research on dietary factors and BMI based on cultural environments are needed. 

#### 2.4.5. Study Limitations

Several potential limitations to this study should be considered. First, this study is a cross-sectional study, which can lead to limited study conclusions given that causation between SES, dietary factors, and behavioral factors and overweight cannot be determined. Therefore, a longitudinal study needs to be carried out in order to confirm the results and the casual relationship between overweight and its risk factors. In addition, data from a longitudinal study could provide important information regarding dietary patterns and food consumption trends and patterns among Thais based on BMI status overtime compared to data from cross-sectional study. Next, this study did not collect data on physical activity or waist circumference, which can be used to assess associated factors of overweight and obesity in this sample. Last, BMI alone may overestimate overweight and obesity in some subgroups and the addition of waist circumference can be used to verify weight status and estimate risk. 

However, this study had several methodological strengths. First, it was conducted in a nationally representative sample covering all geographic regions of Thailand. The data from this study can be used to examine national prevalence estimates for a variety of health issues and provide important insight into the issue of preventing and controlling the excess weight gain. In addition, the sample population in this study was large and included individuals from a variety of age groups. Next, the heights and body weights of participants were actually measured rather than using self-reported values, resulting in much more accurate assessments of BMI than typically found in most population-based studies. 

## 3. Experimental Section

### 3.1. Study Design and Selection Procedures

The current study is a cross-sectional survey design in a representative sample of Thai adults aged 18 old and over, the Thai Food Consumption Survey (TFCS). It was conducted from January 2004 to February 2005 in Thailand using a stratified three-stage sampling design, and was funded by the National Bureau of Agricultural Commodity and Food Standards, Ministry of Agriculture and Cooperative, Thailand. While a wide variety of health issues were assessed in the parent study [46], the primary aim of this study was focused on overweight and obesity prevalence and its risk factors in adult Thais.

Participants were randomly drawn from the local government registers of household lists and only one individual was recruited from a household, without replacement. Eligible participants who were between 18 to 70 years of age, were neither pregnant nor breast-feeding were invited to participate. Pregnant and lactating women were excluded from this study because of differences in food intake and body weight accumulation during pregnancy. In addition, individuals who were older than 70 years were excluded because there are substantial data demonstrating that body mass index (BMI) has been found to be less informative of health risks and mortality among persons aged 70 years and over [47,48,49,50]. For each individual who agreed to participate, the study protocol was described, and an institutionally approved consent form was signed. 

### 3.2. Measurement

Trained staff conducted all assessments including a structured questionnaire and anthropometric measurement at participants’ homes. The questionnaire included basic socio-demographic (such as marital status, education level, occupations and household income) information, cigarette smoking habit, alcohol consumption, and food intake. The socio-demographics (age, education, marital status, household income), behavior characteristic (smoking habit, alcohol consumption), and dietary intake were considered as covariates. 

Education level was divided into three groups based on years of education completed: 1) basic education (1-6 years); 2) secondary education (7-12 years); and 3) higher education (more than 12 years). For occupational status, participants were classified into four occupational groups: 1) managers & professionals; 2) semi-professionals; 3) routine non-manual laborers; and 4) manual laborers. Individuals who were retired, unable to work, students, and housewives were excluded. Participants also were asked to report their household income excluding taxes. Gross household income from all sources was converted from Thai Baht to the U.S. dollars (USD$), and the Thailand poverty line was applied in order to categorize participants into two groups: households that had an annual income below and above poverty line (the recent Thailand poverty line was equivalent to USD$508.1 per year) [51].

Dietary intake was recorded using the 24-hour recall method. All foods and drinks consumed over the previous 24-hour period were recorded. In addition, a Food Frequency Questionnaire (FFQ) was administered. Repeat interviews were conducted in randomly selected sub-samples in order to obtain additional information about dietary intake. Nutrient intake from 24-hour recall and FFQ were entered and verified by another person and analyzed using the specialized Thai software INMUCAL program (Mahidol University, 2006). Carbohydrate, protein, and fat intake were categorized into four groups based on percentage of Thai Recommended Daily Intake (Thai RDI) [52]: 1) less than 100%; 2) 100-150%; 3) 150-200%; and 4) more than 200%. In addition, the consumption of five major food groups (rice and starchy, vegetables, fruits, dairy and meat products) were classified into three groups based on a recommended daily serving size from the Food Guide Thailand Nutrition Flag [53]: 1) less than serving recommendation; 2) twice of the recommendations; and 3) more than two times of the recommendations. 

The physical examination was performed with anthropometric measurements including body weight and height with participants wearing indoor clothes without shoes. BMI was calculated as weight (kg) divided by height squared (m^2^) and was rounded to the nearest 0.1 (kg/m^2^). The Regional Office for the Western Pacific (WPRO) standards were used to categorize adult overweight and obesity [1]. According to the WPRO criteria, BMI 18.5 to 22.9 kg/m^2 ^was classified as normal or healthy weight, and BMI ≥ 23.0 kg/m^2 ^as overweight and obese.

### 3.3. Statistical Analysis

Statistical analyses were performed using SPSS ® (version 16.0; SPSS Inc., Chicago, IL, USA). For the overweight/obesity prevalence data among genders, sample size and percentages were reported and Chi-square was used to test the differences between males and females. Chi-square, *t*-test and ANOVA were applied to examine differences in SES characteristics, behavioral factors, and dietary intakes separated by genders. The associations between being overweight and obese (where 0 = healthy weight and 1 = being overweight/obese) and each of the SES indicators were examined in overall and gender-stratified multivariate binary logistic regression models. For each SES indicator, the least advantaged group was used as the reference group. The results are presented as age-adjusted odds ratios (OR) and their 95% confidence intervals (CI). The significance level was set at *p* < 0.05

## 4. Conclusions

SES status indicators are associated with risk of being overweight and obese in Thai adults, but the associations were different between genders. Education was a strong predictor of overweight and obesity in women, whereas annual household income was significantly associated with a higher BMI in men. Besides the SES factors, smoking habit, carbohydrate and protein intake and dairy product consumption were associated with overweight and obesity among men. Health promotion strategies regarding obesity and its related co-morbidities are necessary. 
